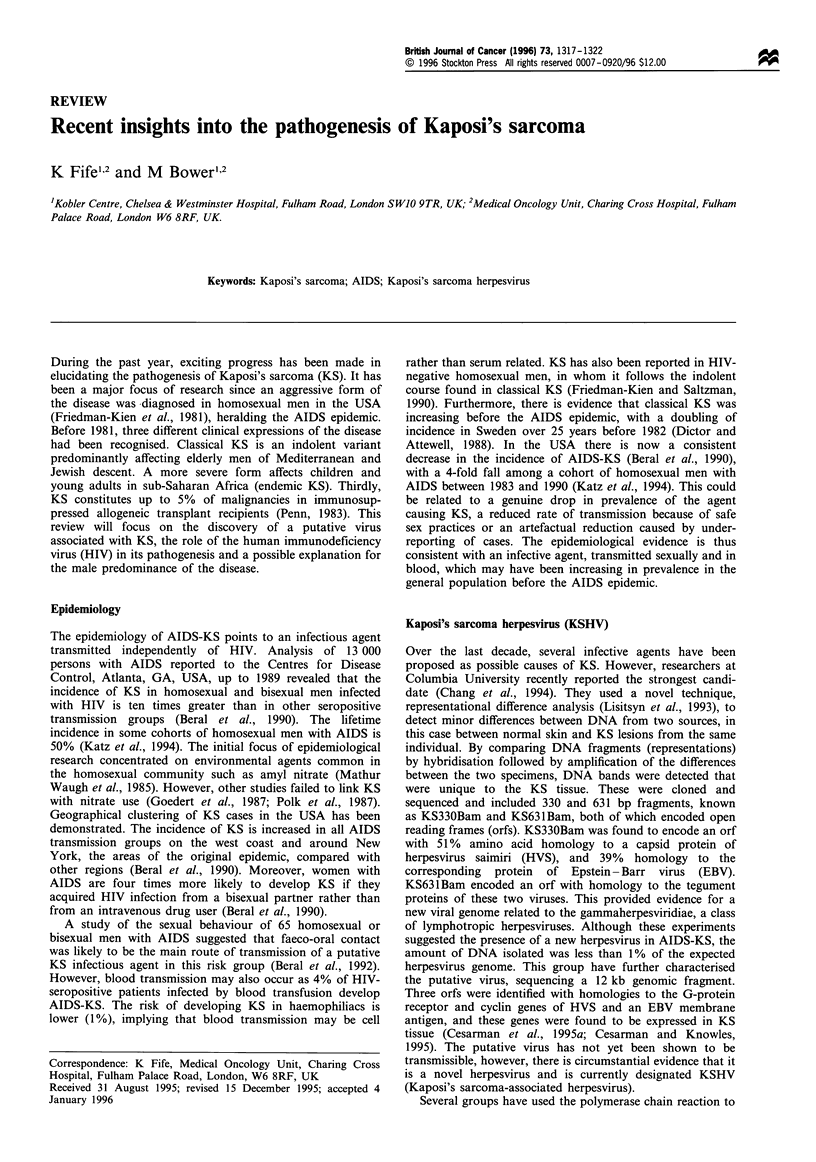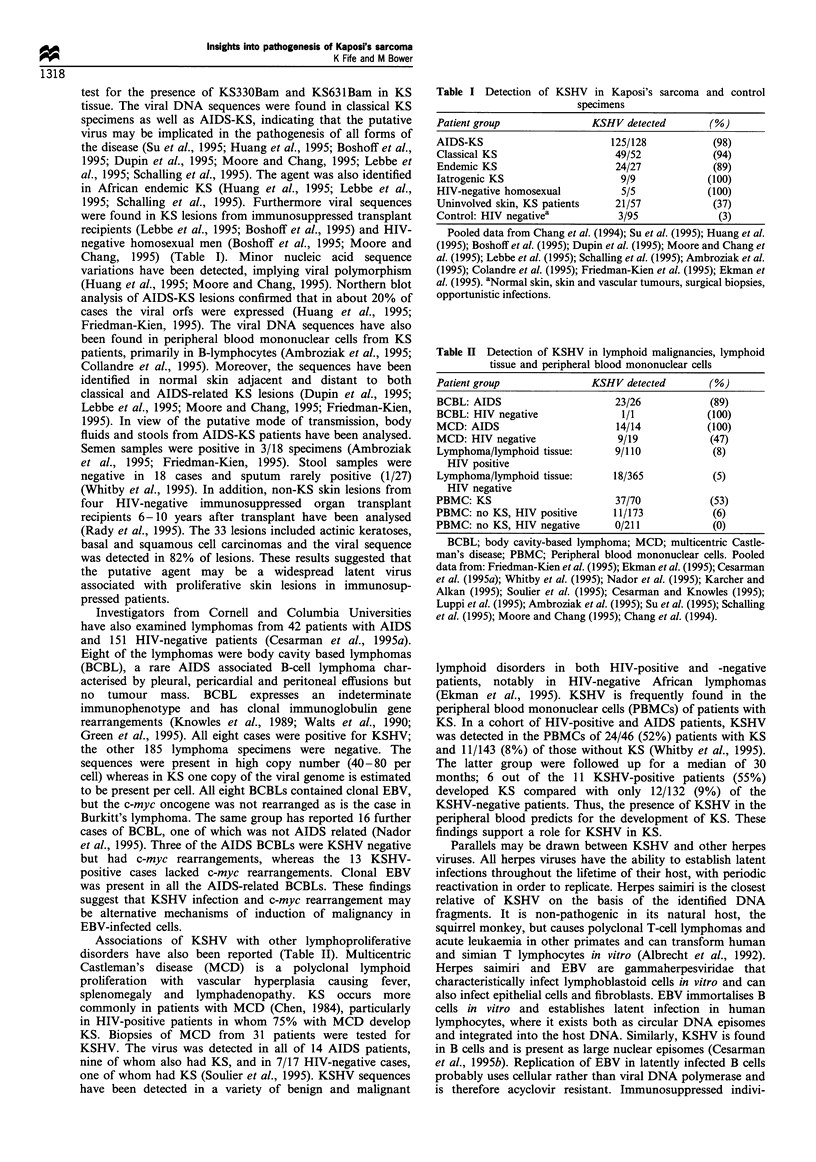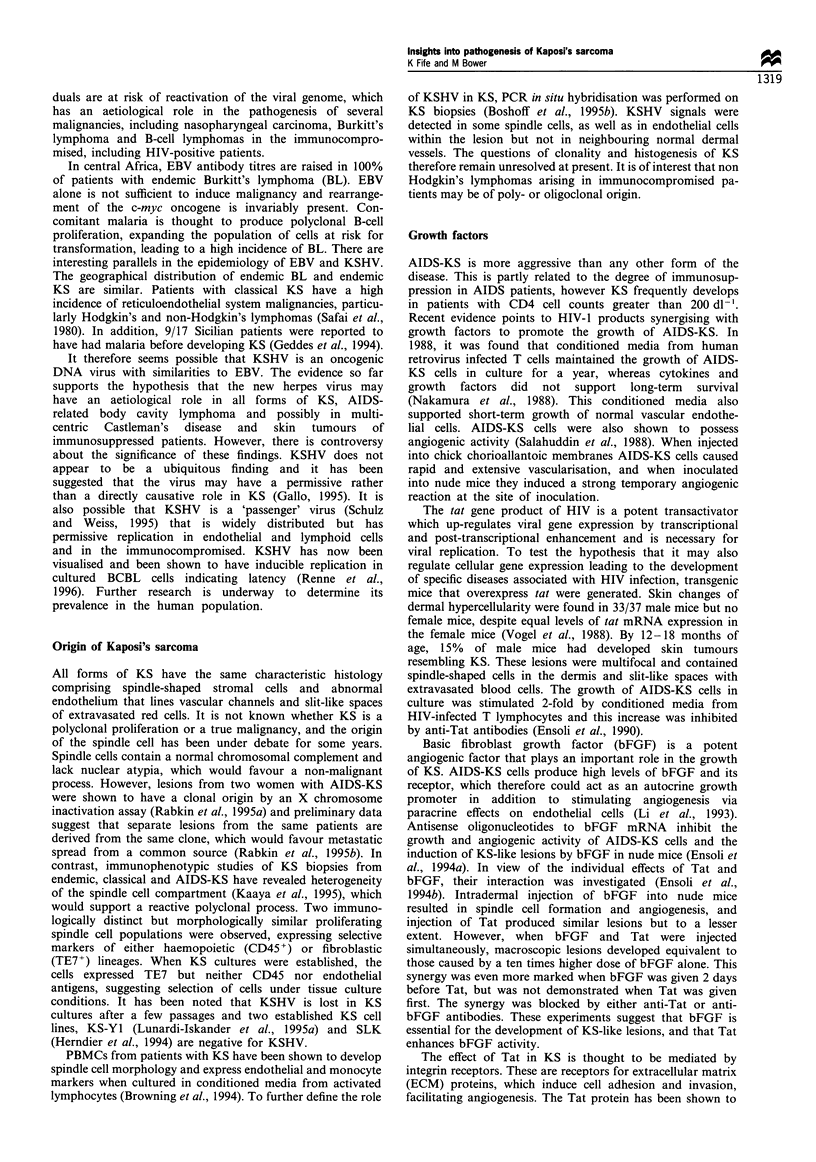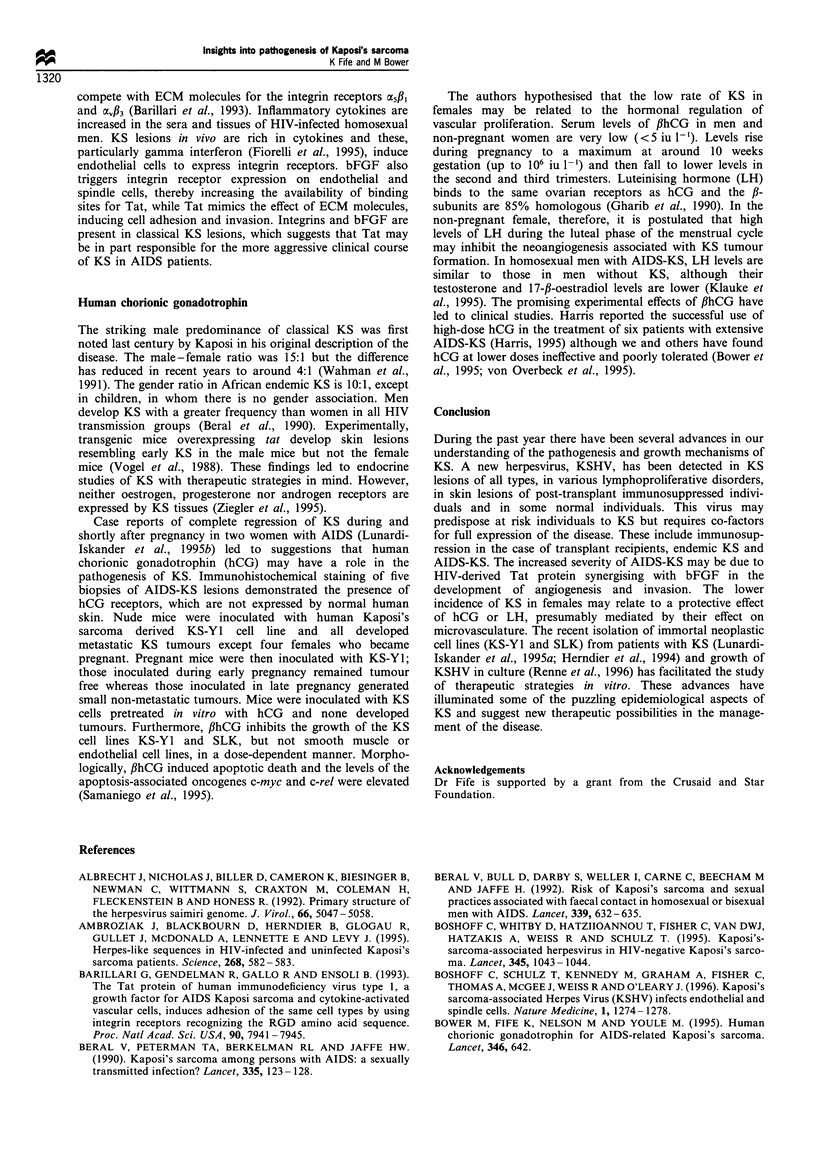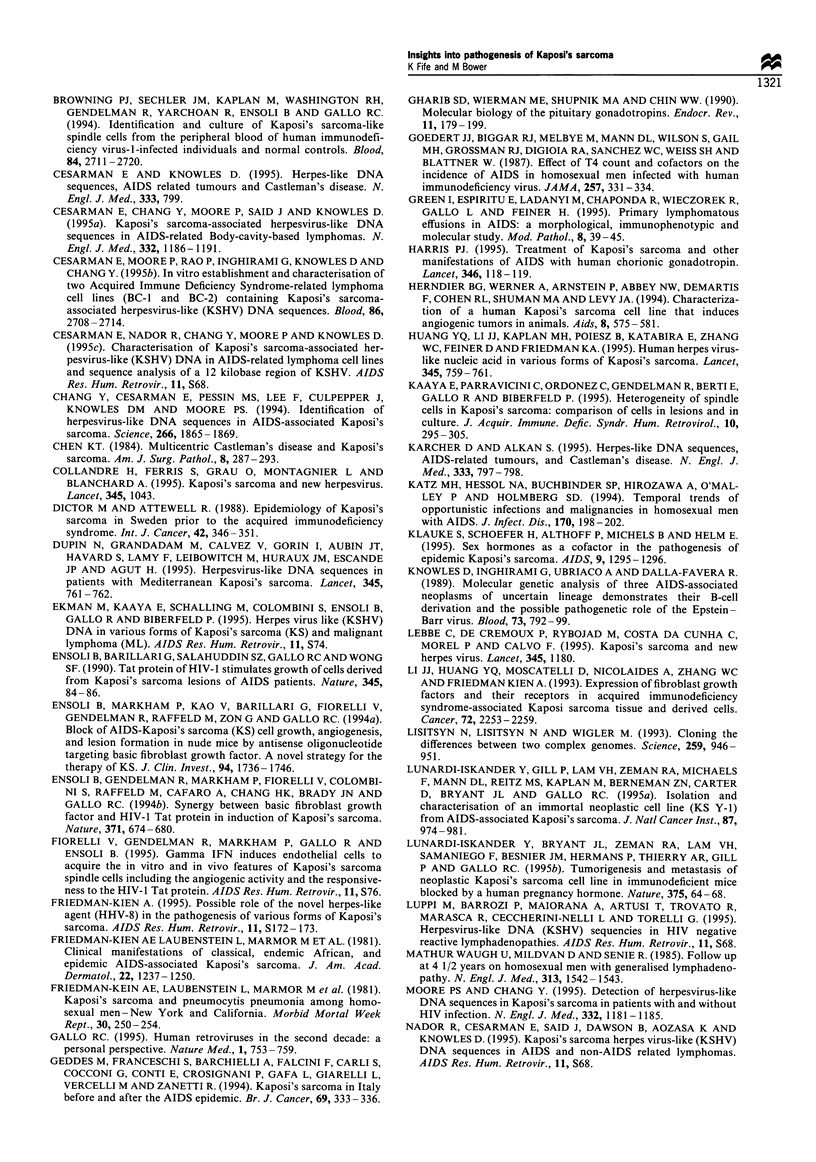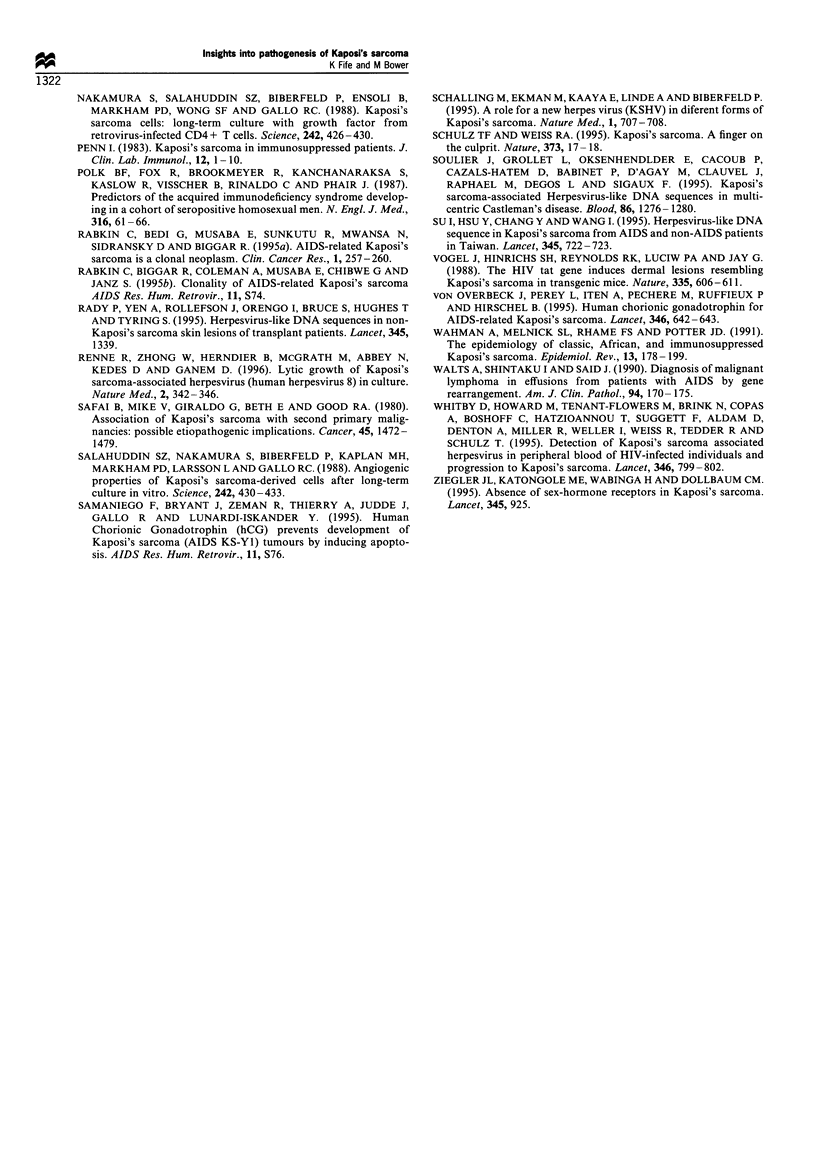# Recent insights into the pathogenesis of Kaposi's sarcoma.

**DOI:** 10.1038/bjc.1996.252

**Published:** 1996-06

**Authors:** K. Fife, M. Bower

**Affiliations:** Kobler Centre, Chelsea & Westminster Hospital, London, UK.


British Journal of Cancer (1996) 73, 1317-1322
© 1996 Stockton Press All rights reserved 0007-0920/96 $12.00

REVIEW

### Recent insights into the pathogenesis of Kaposi's sarcoma

K Fife[1,2] and M Bower[1,2]

[1]Kobler Centre, Chelsea & Westminster Hospital, Fulham Road, London SW10 9TR, UK; [2]Medical Oncology Unit, Charing Cross Hospital, Fulham Palace Road, London W6 8RF, UK.

**Keywords:** Kaposi's sarcoma; AIDS; Kaposi's sarcoma herpesvirus

During the past year, exciting progress has been made in elucidating the pathogenesis of Kaposi's sarcoma (KS). It has been a major focus of research since an aggressive form of the disease was diagnosed in homosexual men in the USA (Friedman-Kien *et al.*, 1981), heralding the AIDS epidemic. Before 1981, three different clinical expressions of the disease had been recognised. Classical KS is an indolent variant predominantly affecting elderly men of Mediterranean and Jewish descent. A more severe form affects children and young adults in sub-Saharan Africa (endemic KS). Thirdly, KS constitutes up to 5% of malignancies in immunosuppressed allogeneic transplant recipients (Penn, 1983). This review will focus on the discovery of a putative virus associated with KS, the role of the human immunodeficiency virus (HIV) in its pathogenesis and a possible explanation for the male predominance of the disease.

### Epidemiology

The epidemiology of AIDS-KS points to an infectious agent transmitted independently of HIV. Analysis of 13 000 persons with AIDS reported to the Centres for Disease Control, Atlanta, GA, USA, up to 1989 revealed that the incidence of KS in homosexual and bisexual men infected with HIV is ten times greater than in other seropositive transmission groups (Beral *et al.*, 1990). The lifetime incidence in some cohorts of homosexual men with AIDS is 50% (Katz *et al.*, 1994). The initial focus of epidemiological research concentrated on environmental agents common in the homosexual community such as amyl nitrate (Mathur Waugh *et al.*, 1985). However, other studies failed to link KS with nitrate use (Goedert *et al.*, 1987; Polk *et al.*, 1987). Geographical clustering of KS cases in the USA has been demonstrated. The incidence of KS is increased in all AIDS transmission groups on the west coast and around New York, the areas of the original epidemic, compared with other regions (Beral *et al.*, 1990). Moreover, women with AIDS are four times more likely to develop KS if they acquired HIV infection from a bisexual partner rather than from an intravenous drug user (Beral *et al.*, 1990).

A study of the sexual behaviour of 65 homosexual or bisexual men with AIDS suggested that faeco-oral contact was likely to be the main route of transmission of a putative KS infectious agent in this risk group (Beral *et al.*, 1992). However, blood transmission may also occur as 4% of HIV-seropositive patients infected by blood transfusion develop AIDS-KS. The risk of developing KS in haemophiliacs is lower (1%), implying that blood transmission may be cell rather than serum related. KS has also been reported in HIV-negative homosexual men, in whom it follows the indolent course found in classical KS (Friedman-Kien and Saltzman, 1990). Furthermore, there is evidence that classical KS was increasing before the AIDS epidemic, with a doubling of incidence in Sweden over 25 years before 1982 (Dictor and Attewell, 1988). In the USA there is now a consistent decrease in the incidence of AIDS-KS (Beral *et al.*, 1990), with a 4-fold fall among a cohort of homosexual men with AIDS between 1983 and 1990 (Katz *et al.*, 1994). This could be related to a genuine drop in prevalence of the agent causing KS, a reduced rate of transmission because of safe sex practices or an artefactual reduction caused by under-reporting of cases. The epidemiological evidence is thus consistent with an infective agent, transmitted sexually and in blood, which may have been increasing in prevalence in the general population before the AIDS epidemic.

### Kaposi's sarcoma herpesvirus (KSHV)

Over the last decade, several infective agents have been proposed as possible causes of KS. However, researchers at Columbia University recently reported the strongest candidate (Chang *et al.*, 1994). They used a novel technique, representational difference analysis (Lisitsyn *et al.*, 1993), to detect minor differences between DNA from two sources, in this case between normal skin and KS lesions from the same individual. By comparing DNA fragments (representations) by hybridisation followed by amplification of the differences between the two specimens, DNA bands were detected that were unique to the KS tissue. These were cloned and sequenced and included 330 and 631 bp fragments, known as KS330Bam and KS631Bam, both of which encoded open reading frames (orfs). KS330Bam was found to encode an orf with 51% amino acid homology to a capsid protein of herpesvirus saimiri (HVS), and 39% homology to the corresponding protein of Epstein–Barr virus (EBV). KS631Bam encoded an orf with homology to the tegument proteins of these two viruses. This provided evidence for a new viral genome related to the gammaherpesviridiae, a class of lymphotropic herpesviruses. Although these experiments suggested the presence of a new herpesvirus in AIDS-KS, the amount of DNA isolated was less than 1% of the expected herpesvirus genome. This group have further characterised the putative virus, sequencing a 12 kb genomic fragment. Three orfs were identified with homologies to the G-protein receptor and cyclin genes of HVS and an EBV membrane antigen, and these genes were found to be expressed in KS tissue (Cesarman *et al.*, 1995*a*; Cesarman and Knowles, 1995). The putative virus has not yet been shown to be transmissible, however, there is circumstantial evidence that it is a novel herpesvirus and is currently designated KSHV (Kaposi's sarcoma-associated herpesvirus).

Several groups have used the polymerase chain reaction to

Correspondence: K Fife, Medical Oncology Unit, Charing Cross Hospital, Fulham Palace Road, London, W6 8RF, UK
Received 31 August 1995; revised 15 December 1995; accepted 4 January 1996

test for the presence of KS330Bam and KS631Bam in KS tissue. The viral DNA sequences were found in classical KS specimens as well as AIDS-KS, indicating that the putative virus may be implicated in the pathogenesis of all forms of the disease (Su *et al.*, 1995; Huang *et al.*, 1995; Boshoff *et al.*, 1995; Dupin *et al.*, 1995; Moore and Chang, 1995; Lebbe *et al.*, 1995; Schalling *et al.*, 1995). The agent was also identified in African endemic KS (Huang *et al.*, 1995; Lebbe *et al.*, 1995; Schalling *et al.*, 1995). Furthermore viral sequences were found in KS lesions from immunosuppressed transplant recipients (Lebbe *et al.*, 1995; Boshoff *et al.*, 1995) and HIV-negative homosexual men (Boshoff *et al.*, 1995; Moore and Chang, 1995) (Table I). Minor nucleic acid sequence variations have been detected, implying viral polymorphism (Huang *et al.*, 1995; Moore and Chang, 1995). Northern blot analysis of AIDS-KS lesions confirmed that in about 20% of cases the viral orfs were expressed (Huang *et al.*, 1995; Friedman-Kien, 1995). The viral DNA sequences have also been found in peripheral blood mononuclear cells from KS patients, primarily in B-lymphocytes (Ambroziak *et al.*, 1995; Collandre *et al.*, 1995). Moreover, the sequences have been identified in normal skin adjacent and distant to both classical and AIDS-related KS lesions (Dupin *et al.*, 1995; Lebbe *et al.*, 1995; Moore and Chang, 1995; Friedman-Kien, 1995). In view of the putative mode of transmission, body fluids and stools from AIDS-KS patients have been analysed. Semen samples were positive in 3/18 specimens (Ambroziak *et al.*, 1995; Friedman-Kien, 1995). Stool samples were negative in 18 cases and sputum rarely positive (1/27) (Whitby *et al.*, 1995). In addition, non-KS skin lesions from four HIV-negative immunosuppressed organ transplant recipients 6–10 years after transplant have been analysed (Rady *et al.*, 1995). The 33 lesions included actinic keratoses, basal and squamous cell carcinomas and the viral sequence was detected in 82% of lesions. These results suggested that the putative agent may be a widespread latent virus associated with proliferative skin lesions in immunosuppressed patients.

Investigators from Cornell and Columbia Universities have also examined lymphomas from 42 patients with AIDS and 151 HIV-negative patients (Cesarman *et al.*, 1995*a*). Eight of the lymphomas were body cavity based lymphomas (BCBL), a rare AIDS associated B-cell lymphoma characterised by pleural, pericardial and peritoneal effusions but no tumour mass. BCBL expresses an indeterminate immunophenotype and has clonal immunoglobulin gene rearrangements (Knowles *et al.*, 1989; Walts *et al.*, 1990; Green *et al.*, 1995). All eight cases were positive for KSHV; the other 185 lymphoma specimens were negative. The sequences were present in high copy number (40–80 per cell) whereas in KS one copy of the viral genome is estimated to be present per cell. All eight BCBLs contained clonal EBV, but the c-*myc* oncogene was not rearranged as is the case in Burkitt's lymphoma. The same group has reported 16 further cases of BCBL, one of which was not AIDS related (Nador *et al.*, 1995). Three of the AIDS BCBLs were KSHV negative but had c-*myc* rearrangements, whereas the 13 KSHV-positive cases lacked c-*myc* rearrangements. Clonal EBV was present in all the AIDS-related BCBLs. These findings suggest that KSHV infection and c-*myc* rearrangement may be alternative mechanisms of induction of malignancy in EBV-infected cells.

Associations of KSHV with other lymphoproliferative disorders have also been reported (Table II). Multicentric Castleman's disease (MCD) is a polyclonal lymphoid proliferation with vascular hyperplasia causing fever, splenomegaly and lymphadenopathy. KS occurs more commonly in patients with MCD (Chen, 1984), particularly in HIV-positive patients in whom 75% with MCD develop KS. Biopsies of MCD from 31 patients were tested for KSHV. The virus was detected in all of 14 AIDS patients, nine of whom also had KS, and in 7/17 HIV-negative cases, one of whom had KS (Soulier *et al.*, 1995). KSHV sequences have been detected in a variety of benign and malignant lymphoid disorders in both HIV-positive and -negative patients, notably in HIV-negative African lymphomas (Ekman *et al.*, 1995). KSHV is frequently found in the peripheral blood mononuclear cells (PBMCs) of patients with KS. In a cohort of HIV-positive and AIDS patients, KSHV was detected in the PBMCs of 24/46 (52%) patients with KS and 11/143 (8%) of those without KS (Whitby *et al.*, 1995). The latter group were followed up for a median of 30 months; 6 out of the 11 KSHV-positive patients (55%) developed KS compared with only 12/132 (9%) of the KSHV-negative patients. Thus, the presence of KSHV in the peripheral blood predicts for the development of KS. These findings support a role for KSHV in KS.

Parallels may be drawn between KSHV and other herpes viruses. All herpes viruses have the ability to establish latent infections throughout the lifetime of their host, with periodic reactivation in order to replicate. Herpes saimiri is the closest relative of KSHV on the basis of the identified DNA fragments. It is non-pathogenic in its natural host, the squirrel monkey, but causes polyclonal T-cell lymphomas and acute leukaemia in other primates and can transform human and simian T lymphocytes *in vitro* (Albrecht *et al.*, 1992). Herpes saimiri and EBV are gammaherpesviridae that characteristically infect lymphoblastoid cells *in vitro* and can also infect epithelial cells and fibroblasts. EBV immortalises B cells *in vitro* and establishes latent infection in human lymphocytes, where it exists both as circular DNA episomes and integrated into the host DNA. Similarly, KSHV is found in B cells and is present as large nuclear episomes (Cesarman *et al.*, 1995*b*). Replication of EBV in latently infected B cells probably uses cellular rather than viral DNA polymerase and is therefore acyclovir resistant. Immunosuppressed indivi-

**Table I** Detection of KSHV in Kaposi's sarcoma and control specimens

| *Patient group* | *KSHV detected* | *(%)* |
|---|---|---|
| AIDS-KS | 125/128 | (98) |
| Classical KS | 49/52 | (94) |
| Endemic KS | 24/27 | (89) |
| Iatrogenic KS | 9/9 | (100) |
| HIV-negative homosexual | 5/5 | (100) |
| Uninvolved skin, KS patients | 21/57 | (37) |
| Control: HIV negative[a] | 3/95 | (3) |

Pooled data from Chang *et al.* (1994); Su *et al.* (1995); Huang *et al.* (1995); Boshoff *et al.* (1995); Dupin *et al.* (1995); Moore and Chang *et al.* (1995); Lebbe *et al.* (1995); Schalling *et al.* (1995); Ambroziak *et al.* (1995); Colandre *et al.* (1995); Friedman-Kien *et al.* (1995); Ekman *et al.* (1995). [a]Normal skin, skin and vascular tumours, surgical biopsies, opportunistic infections.

**Table II** Detection of KSHV in lymphoid malignancies, lymphoid tissue and peripheral blood mononuclear cells

| *Patient group* | *KSHV detected* | *(%)* |
|---|---|---|
| BCBL: AIDS | 23/26 | (89) |
| BCBL: HIV negative | 1/1 | (100) |
| MCD: AIDS | 14/14 | (100) |
| MCD: HIV negative | 9/19 | (47) |
| Lymphoma/lymphoid tissue: HIV positive | 9/110 | (8) |
| Lymphoma/lymphoid tissue: HIV negative | 18/365 | (5) |
| PBMC: KS | 37/70 | (53) |
| PBMC: no KS, HIV positive | 11/173 | (6) |
| PBMC: no KS, HIV negative | 0/211 | (0) |

BCBL; body cavity-based lymphoma; MCD; multicentric Castleman's disease; PBMC; Peripheral blood mononuclear cells. Pooled data from: Friedman-Kien *et al.* (1995); Ekman *et al.* (1995); Cesarman *et al.* (1995*a*); Whitby *et al.* (1995); Nador *et al.* (1995); Karcher and Alkan (1995); Soulier *et al.* (1995); Cesarman and Knowles (1995); Luppi *et al.* (1995); Ambroziak *et al.* (1995); Su *et al.* (1995); Schalling *et al.* (1995); Moore and Chang (1995); Chang *et al.* (1994).

duals are at risk of reactivation of the viral genome, which has an aetiological role in the pathogenesis of several malignancies, including nasopharyngeal carcinoma, Burkitt's lymphoma and B-cell lymphomas in the immunocompromised, including HIV-positive patients.

In central Africa, EBV antibody titres are raised in 100% of patients with endemic Burkitt's lymphoma (BL). EBV alone is not sufficient to induce malignancy and rearrangement of the c-*myc* oncogene is invariably present. Concomitant malaria is thought to produce polyclonal B-cell proliferation, expanding the population of cells at risk for transformation, leading to a high incidence of BL. There are interesting parallels in the epidemiology of EBV and KSHV. The geographical distribution of endemic BL and endemic KS are similar. Patients with classical KS have a high incidence of reticuloendothelial system malignancies, particularly Hodgkin's and non-Hodgkin's lymphomas (Safai *et al.*, 1980). In addition, 9/17 Sicilian patients were reported to have had malaria before developing KS (Geddes *et al.*, 1994).

It therefore seems possible that KSHV is an oncogenic DNA virus with similarities to EBV. The evidence so far supports the hypothesis that the new herpes virus may have an aetiological role in all forms of KS, AIDS-related body cavity lymphoma and possibly in multicentric Castleman's disease and skin tumours of immunosuppressed patients. However, there is controversy about the significance of these findings. KSHV does not appear to be a ubiquitous finding and it has been suggested that the virus may have a permissive rather than a directly causative role in KS (Gallo, 1995). It is also possible that KSHV is a 'passenger' virus (Schulz and Weiss, 1995) that is widely distributed but has permissive replication in endothelial and lymphoid cells and in the immunocompromised. KSHV has now been visualised and been shown to have inducible replication in cultured BCBL cells indicating latency (Renne *et al.*, 1996). Further research is underway to determine its prevalence in the human population.

### Origin of Kaposi's sarcoma

All forms of KS have the same characteristic histology comprising spindle-shaped stromal cells and abnormal endothelium that lines vascular channels and slit-like spaces of extravasated red cells. It is not known whether KS is a polyclonal proliferation or a true malignancy, and the origin of the spindle cell has been under debate for some years. Spindle cells contain a normal chromosomal complement and lack nuclear atypia, which would favour a non-malignant process. However, lesions from two women with AIDS-KS were shown to have a clonal origin by an X chromosome inactivation assay (Rabkin *et al.*, 1995*a*) and preliminary data suggest that separate lesions from the same patients are derived from the same clone, which would favour metastatic spread from a common source (Rabkin *et al.*, 1995*b*). In contrast, immunophenotypic studies of KS biopsies from endemic, classical and AIDS-KS have revealed heterogeneity of the spindle cell compartment (Kaaya *et al.*, 1995), which would support a reactive polyclonal process. Two immunologically distinct but morphologically similar proliferating spindle cell populations were observed, expressing selective markers of either haemopoietic ($CD45^+$) or fibroblastic ($TE7^+$) lineages. When KS cultures were established, the cells expressed TE7 but neither CD45 nor endothelial antigens, suggesting selection of cells under tissue culture conditions. It has been noted that KSHV is lost in KS cultures after a few passages and two established KS cell lines, KS-Y1 (Lunardi-Iskander *et al.*, 1995*a*) and SLK (Herndier *et al.*, 1994) are negative for KSHV.

PBMCs from patients with KS have been shown to develop spindle cell morphology and express endothelial and monocyte markers when cultured in conditioned media from activated lymphocytes (Browning *et al.*, 1994). To further define the role of KSHV in KS, PCR *in situ* hybridisation was performed on KS biopsies (Boshoff *et al.*, 1995*b*). KSHV signals were detected in some spindle cells, as well as in endothelial cells within the lesion but not in neighbouring normal dermal vessels. The questions of clonality and histogenesis of KS therefore remain unresolved at present. It is of interest that non Hodgkin's lymphomas arising in immunocompromised patients may be of poly- or oligoclonal origin.

### Growth factors

AIDS-KS is more aggressive than any other form of the disease. This is partly related to the degree of immunosuppression in AIDS patients, however KS frequently develops in patients with CD4 cell counts greater than 200 $dl^{-1}$. Recent evidence points to HIV-1 products synergising with growth factors to promote the growth of AIDS-KS. In 1988, it was found that conditioned media from human retrovirus infected T cells maintained the growth of AIDS-KS cells in culture for a year, whereas cytokines and growth factors did not support long-term survival (Nakamura *et al.*, 1988). This conditioned media also supported short-term growth of normal vascular endothelial cells. AIDS-KS cells were also shown to possess angiogenic activity (Salahuddin *et al.*, 1988). When injected into chick chorioallantoic membranes AIDS-KS cells caused rapid and extensive vascularisation, and when inoculated into nude mice they induced a strong temporary angiogenic reaction at the site of inoculation.

The *tat* gene product of HIV is a potent transactivator which up-regulates viral gene expression by transcriptional and post-transcriptional enhancement and is necessary for viral replication. To test the hypothesis that it may also regulate cellular gene expression leading to the development of specific diseases associated with HIV infection, transgenic mice that overexpress *tat* were generated. Skin changes of dermal hypercellularity were found in 33/37 male mice but no female mice, despite equal levels of *tat* mRNA expression in the female mice (Vogel *et al.*, 1988). By 12–18 months of age, 15% of male mice had developed skin tumours resembling KS. These lesions were multifocal and contained spindle-shaped cells in the dermis and slit-like spaces with extravasated blood cells. The growth of AIDS-KS cells in culture was stimulated 2-fold by conditioned media from HIV-infected T lymphocytes and this increase was inhibited by anti-Tat antibodies (Ensoli *et al.*, 1990).

Basic fibroblast growth factor (bFGF) is a potent angiogenic factor that plays an important role in the growth of KS. AIDS-KS cells produce high levels of bFGF and its receptor, which therefore could act as an autocrine growth promoter in addition to stimulating angiogenesis via paracrine effects on endothelial cells (Li *et al.*, 1993). Antisense oligonucleotides to bFGF mRNA inhibit the growth and angiogenic activity of AIDS-KS cells and the induction of KS-like lesions by bFGF in nude mice (Ensoli *et al.*, 1994*a*). In view of the individual effects of Tat and bFGF, their interaction was investigated (Ensoli *et al.*, 1994*b*). Intradermal injection of bFGF into nude mice resulted in spindle cell formation and angiogenesis, and injection of Tat produced similar lesions but to a lesser extent. However, when bFGF and Tat were injected simultaneously, macroscopic lesions developed equivalent to those caused by a ten times higher dose of bFGF alone. This synergy was even more marked when bFGF was given 2 days before Tat, but was not demonstrated when Tat was given first. The synergy was blocked by either anti-Tat or anti-bFGF antibodies. These experiments suggest that bFGF is essential for the development of KS-like lesions, and that Tat enhances bFGF activity.

The effect of Tat in KS is thought to be mediated by integrin receptors. These are receptors for extracellular matrix (ECM) proteins, which induce cell adhesion and invasion, facilitating angiogenesis. The Tat protein has been shown to compete with ECM molecules for the integrin receptors $\alpha_5\beta_1$ and $\alpha_v\beta_3$ (Barillari *et al.*, 1993). Inflammatory cytokines are increased in the sera and tissues of HIV-infected homosexual men. KS lesions *in vivo* are rich in cytokines and these, particularly gamma interferon (Fiorelli *et al.*, 1995), induce endothelial cells to express integrin receptors. bFGF also triggers integrin receptor expression on endothelial and spindle cells, thereby increasing the availability of binding sites for Tat, while Tat mimics the effect of ECM molecules, inducing cell adhesion and invasion. Integrins and bFGF are present in classical KS lesions, which suggests that Tat may be in part responsible for the more aggressive clinical course of KS in AIDS patients.

### Human chorionic gonadotrophin

The striking male predominance of classical KS was first noted last century by Kaposi in his original description of the disease. The male–female ratio was 15:1 but the difference has reduced in recent years to around 4:1 (Wahman *et al.*, 1991). The gender ratio in African endemic KS is 10:1, except in children, in whom there is no gender association. Men develop KS with a greater frequency than women in all HIV transmission groups (Beral *et al.*, 1990). Experimentally, transgenic mice overexpressing *tat* develop skin lesions resembling early KS in the male mice but not the female mice (Vogel *et al.*, 1988). These findings led to endocrine studies of KS with therapeutic strategies in mind. However, neither oestrogen, progesterone nor androgen receptors are expressed by KS tissues (Ziegler *et al.*, 1995).

Case reports of complete regression of KS during and shortly after pregnancy in two women with AIDS (Lunardi-Iskander *et al.*, 1995*b*) led to suggestions that human chorionic gonadotrophin (hCG) may have a role in the pathogenesis of KS. Immunohistochemical staining of five biopsies of AIDS-KS lesions demonstrated the presence of hCG receptors, which are not expressed by normal human skin. Nude mice were inoculated with human Kaposi's sarcoma derived KS-Y1 cell line and all developed metastatic KS tumours except four females who became pregnant. Pregnant mice were then inoculated with KS-Y1; those inoculated during early pregnancy remained tumour free whereas those inoculated in late pregnancy generated small non-metastatic tumours. Mice were inoculated with KS cells pretreated *in vitro* with hCG and none developed tumours. Furthermore, $\beta$hCG inhibits the growth of the KS cell lines KS-Y1 and SLK, but not smooth muscle or endothelial cell lines, in a dose-dependent manner. Morphologically, $\beta$hCG induced apoptotic death and the levels of the apoptosis-associated oncogenes c-*myc* and c-*rel* were elevated (Samaniego *et al.*, 1995).

The authors hypothesised that the low rate of KS in females may be related to the hormonal regulation of vascular proliferation. Serum levels of $\beta$hCG in men and non-pregnant women are very low ($< 5$ iu $l^{-1}$). Levels rise during pregnancy to a maximum at around 10 weeks gestation (up to $10^6$ iu $l^{-1}$) and then fall to lower levels in the second and third trimesters. Luteinising hormone (LH) binds to the same ovarian receptors as hCG and the $\beta$-subunits are 85% homologous (Gharib *et al.*, 1990). In the non-pregnant female, therefore, it is postulated that high levels of LH during the luteal phase of the menstrual cycle may inhibit the neoangiogenesis associated with KS tumour formation. In homosexual men with AIDS-KS, LH levels are similar to those in men without KS, although their testosterone and 17-$\beta$-oestradiol levels are lower (Klauke *et al.*, 1995). The promising experimental effects of $\beta$hCG have led to clinical studies. Harris reported the successful use of high-dose hCG in the treatment of six patients with extensive AIDS-KS (Harris, 1995) although we and others have found hCG at lower doses ineffective and poorly tolerated (Bower *et al.*, 1995; von Overbeck *et al.*, 1995).

### Conclusion

During the past year there have been several advances in our understanding of the pathogenesis and growth mechanisms of KS. A new herpesvirus, KSHV, has been detected in KS lesions of all types, in various lymphoproliferative disorders, in skin lesions of post-transplant immunosuppressed individuals and in some normal individuals. This virus may predispose at risk individuals to KS but requires co-factors for full expression of the disease. These include immunosuppression in the case of transplant recipients, endemic KS and AIDS-KS. The increased severity of AIDS-KS may be due to HIV-derived Tat protein synergising with bFGF in the development of angiogenesis and invasion. The lower incidence of KS in females may relate to a protective effect of hCG or LH, presumably mediated by their effect on microvasculature. The recent isolation of immortal neoplastic cell lines (KS-Y1 and SLK) from patients with KS (Lunardi-Iskander *et al.*, 1995*a*; Herndier *et al.*, 1994) and growth of KSHV in culture (Renne *et al.*, 1996) has facilitated the study of therapeutic strategies *in vitro*. These advances have illuminated some of the puzzling epidemiological aspects of KS and suggest new therapeutic possibilities in the management of the disease.

**Acknowledgements**
Dr Fife is supported by a grant from the Crusaid and Star Foundation.

### References

ALBRECHT J, NICHOLAS J, BILLER D, CAMERON K, BIESINGER B, NEWMAN C, WITTMANN S, CRAXTON M, COLEMAN H, FLECKENSTEIN B AND HONESS R. (1992). Primary structure of the herpesvirus saimiri genome. *J. Virol.*, **66**, 5047–5058.

AMBROZIAK J, BLACKBOURN D, HERNDIER B, GLOGAU R, GULLET J, MCDONALD A, LENNETTE E AND LEVY J. (1995). Herpes-like sequences in HIV-infected and uninfected Kaposi's sarcoma patients. *Science*, **268**, 582–583.

BARILLARI G, GENDELMAN R, GALLO R AND ENSOLI B. (1993). The Tat protein of human immunodeficiency virus type 1, a growth factor for AIDS Kaposi sarcoma and cytokine-activated vascular cells, induces adhesion of the same cell types by using integrin receptors recognizing the RGD amino acid sequence. *Proc. Natl Acad. Sci. USA*, **90**, 7941–7945.

BERAL V, PETERMAN TA, BERKELMAN RL AND JAFFE HW. (1990). Kaposi's sarcoma among persons with AIDS: a sexually transmitted infection? *Lancet*, **335**, 123–128.

BERAL V, BULL D, DARBY S, WELLER I, CARNE C, BEECHAM M AND JAFFE H. (1992). Risk of Kaposi's sarcoma and sexual practices associated with faecal contact in homosexual or bisexual men with AIDS. *Lancet*, **339**, 632–635.

BOSHOFF C, WHITBY D, HATZIIOANNOU T, FISHER C, VAN DWJ, HATZAKIS A, WEISS R AND SCHULZ T. (1995). Kaposi's-sarcoma-associated herpesvirus in HIV-negative Kaposi's sarcoma. *Lancet*, **345**, 1043–1044.

BOSHOFF C, SCHULZ T, KENNEDY M, GRAHAM A, FISHER C, THOMAS A, MCGEE J, WEISS R AND O'LEARY J. (1996). Kaposi's sarcoma-associated Herpes Virus (KSHV) infects endothelial and spindle cells. *Nature Medicine*, **1**, 1274–1278.

BOWER M, FIFE K, NELSON M AND YOULE M. (1995). Human chorionic gonadotrophin for AIDS-related Kaposi's sarcoma. *Lancet*, **346**, 642.

BROWNING PJ, SECHLER JM, KAPLAN M, WASHINGTON RH, GENDELMAN R, YARCHOAN R, ENSOLI B AND GALLO RC. (1994). Identification and culture of Kaposi's sarcoma-like spindle cells from the peripheral blood of human immunodeficiency virus-1-infected individuals and normal controls. *Blood*, **84**, 2711–2720.

CESARMAN E AND KNOWLES D. (1995). Herpes-like DNA sequences, AIDS related tumours and Castleman's disease. *N. Engl. J. Med.*, **333**, 799.

CESARMAN E, CHANG Y, MOORE P, SAID J AND KNOWLES D. (1995*a*). Kaposi's sarcoma-associated herpesvirus-like DNA sequences in AIDS-related Body-cavity-based lymphomas. *N. Engl. J. Med.*, **332**, 1186–1191.

CESARMAN E, MOORE P, RAO P, INGHIRAMI G, KNOWLES D AND CHANG Y. (1995*b*). In vitro establishment and characterisation of two Acquired Immune Deficiency Syndrome-related lymphoma cell lines (BC-1 and BC-2) containing Kaposi's sarcoma-associated herpesvirus-like (KSHV) DNA sequences. *Blood*, **86**, 2708–2714.

CESARMAN E, NADOR R, CHANG Y, MOORE P AND KNOWLES D. (1995*c*). Characterisation of Kaposi's sarcoma-associated herpesvirus-like (KSHV) DNA in AIDS-related lymphoma cell lines and sequence analysis of a 12 kilobase region of KSHV. *AIDS Res. Hum. Retrovir.*, **11**, S68.

CHANG Y, CESARMAN E, PESSIN MS, LEE F, CULPEPPER J, KNOWLES DM AND MOORE PS. (1994). Identification of herpesvirus-like DNA sequences in AIDS-associated Kaposi's sarcoma. *Science*, **266**, 1865–1869.

CHEN KT. (1984). Multicentric Castleman's disease and Kaposi's sarcoma. *Am. J. Surg. Pathol.*, **8**, 287–293.

COLLANDRE H, FERRIS S, GRAU O, MONTAGNIER L AND BLANCHARD A. (1995). Kaposi's sarcoma and new herpesvirus. *Lancet*, **345**, 1043.

DICTOR M AND ATTEWELL R. (1988). Epidemiology of Kaposi's sarcoma in Sweden prior to the acquired immunodeficiency syndrome. *Int. J. Cancer*, **42**, 346–351.

DUPIN N, GRANDADAM M, CALVEZ V, GORIN I, AUBIN JT, HAVARD S, LAMY F, LEIBOWITCH M, HURAUX JM, ESCANDE JP AND AGUT H. (1995). Herpesvirus-like DNA sequences in patients with Mediterranean Kaposi's sarcoma. *Lancet*, **345**, 761–762.

EKMAN M, KAAYA E, SCHALLING M, COLOMBINI S, ENSOLI B, GALLO R AND BIBERFELD P. (1995). Herpes virus like (KSHV) DNA in various forms of Kaposi's sarcoma (KS) and malignant lymphoma (ML). *AIDS Res. Hum. Retrovir.*, **11**, S74.

ENSOLI B, BARILLARI G, SALAHUDDIN SZ, GALLO RC AND WONG SF. (1990). Tat protein of HIV-1 stimulates growth of cells derived from Kaposi's sarcoma lesions of AIDS patients. *Nature*, **345**, 84–86.

ENSOLI B, MARKHAM P, KAO V, BARILLARI G, FIORELLI V, GENDELMAN R, RAFFELD M, ZON G AND GALLO RC. (1994*a*). Block of AIDS-Kaposi's sarcoma (KS) cell growth, angiogenesis, and lesion formation in nude mice by antisense oligonucleotide targeting basic fibroblast growth factor. A novel strategy for the therapy of KS. *J. Clin. Invest.*, **94**, 1736–1746.

ENSOLI B, GENDELMAN R, MARKHAM P, FIORELLI V, COLOMBINI S, RAFFELD M, CAFARO A, CHANG HK, BRADY JN AND GALLO RC. (1994*b*). Synergy between basic fibroblast growth factor and HIV-1 Tat protein in induction of Kaposi's sarcoma. *Nature*, **371**, 674–680.

FIORELLI V, GENDELMAN R, MARKHAM P, GALLO R AND ENSOLI B. (1995). Gamma IFN induces endothelial cells to acquire the in vitro and in vivo features of Kaposi's sarcoma spindle cells including the angiogenic activity and the responsiveness to the HIV-1 Tat protein. *AIDS Res. Hum. Retrovir.*, **11**, S76.

FRIEDMAN-KIEN A. (1995). Possible role of the novel herpes-like agent (HHV-8) in the pathogenesis of various forms of Kaposi's sarcoma. *AIDS Res. Hum. Retrovir.*, **11**, S172–173.

FRIEDMAN-KIEN AE LAUBENSTEIN L, MARMOR M ET AL. (1981). Clinical manifestations of classical, endemic African, and epidemic AIDS-associated Kaposi's sarcoma. *J. Am. Acad. Dermatol.*, **22**, 1237–1250.

FRIEDMAN-KEIN AE, LAUBENSTEIN L, MARMOR M *et al.* (1981). Kaposi's sarcoma and pneumocytis pneumonia among homosexual men–New York and California. *Morbid Mortal Week Rept.*, **30**, 250–254.

GALLO RC. (1995). Human retroviruses in the second decade: a personal perspective. *Nature Med.*, **1**, 753–759.

GEDDES M, FRANCESCHI S, BARCHIELLI A, FALCINI F, CARLI S, COCCONI G, CONTI E, CROSIGNANI P, GAFA L, GIARELLI L, VERCELLI M AND ZANETTI R. (1994). Kaposi's sarcoma in Italy before and after the AIDS epidemic. *Br. J. Cancer*, **69**, 333–336.

GHARIB SD, WIERMAN ME, SHUPNIK MA AND CHIN WW. (1990). Molecular biology of the pituitary gonadotropins. *Endocr. Rev.*, **11**, 179–199.

GOEDERT JJ, BIGGAR RJ, MELBYE M, MANN DL, WILSON S, GAIL MH, GROSSMAN RJ, DIGIOIA RA, SANCHEZ WC, WEISS SH AND BLATTNER W. (1987). Effect of T4 count and cofactors on the incidence of AIDS in homosexual men infected with human immunodeficiency virus. *JAMA*, **257**, 331–334.

GREEN I, ESPIRITU E, LADANYI M, CHAPONDA R, WIECZOREK R, GALLO L AND FEINER H. (1995). Primary lymphomatous effusions in AIDS: a morphological, immunophenotypic and molecular study. *Mod. Pathol.*, **8**, 39–45.

HARRIS PJ. (1995). Treatment of Kaposi's sarcoma and other manifestations of AIDS with human chorionic gonadotropin. *Lancet*, **346**, 118–119.

HERNDIER BG, WERNER A, ARNSTEIN P, ABBEY NW, DEMARTIS F, COHEN RL, SHUMAN MA AND LEVY JA. (1994). Characterization of a human Kaposi's sarcoma cell line that induces angiogenic tumors in animals. *Aids*, **8**, 575–581.

HUANG YQ, LI JJ, KAPLAN MH, POIESZ B, KATABIRA E, ZHANG WC, FEINER D AND FRIEDMAN KA. (1995). Human herpes virus-like nucleic acid in various forms of Kaposi's sarcoma. *Lancet*, **345**, 759–761.

KAAYA E, PARRAVICINI C, ORDONEZ C, GENDELMAN R, BERTI E, GALLO R AND BIBERFELD P. (1995). Heterogeneity of spindle cells in Kaposi's sarcoma: comparison of cells in lesions and in culture. *J. Acquir. Immune. Defic. Syndr. Hum. Retrovirol.*, **10**, 295–305.

KARCHER D AND ALKAN S. (1995). Herpes-like DNA sequences, AIDS-related tumours, and Castleman's disease. *N. Engl. J. Med.*, **333**, 797–798.

KATZ MH, HESSOL NA, BUCHBINDER SP, HIROZAWA A, O'MALLEY P AND HOLMBERG SD. (1994). Temporal trends of opportunistic infections and malignancies in homosexual men with AIDS. *J. Infect. Dis.*, **170**, 198–202.

KLAUKE S, SCHOEFER H, ALTHOFF P, MICHELS B AND HELM E. (1995). Sex hormones as a cofactor in the pathogenesis of epidemic Kaposi's sarcoma. *AIDS*, **9**, 1295–1296.

KNOWLES D, INGHIRAMI G, UBRIACO A AND DALLA-FAVERA R. (1989). Molecular genetic analysis of three AIDS-associated neoplasms of uncertain lineage demonstrates their B-cell derivation and the possible pathogenetic role of the Epstein–Barr virus. *Blood*, **73**, 792–99.

LEBBE C, DE CREMOUX P, RYBOJAD M, COSTA DA CUNHA C, MOREL P AND CALVO F. (1995). Kaposi's sarcoma and new herpes virus. *Lancet*, **345**, 1180.

LI JJ, HUANG YQ, MOSCATELLI D, NICOLAIDES A, ZHANG WC AND FRIEDMAN KIEN A. (1993). Expression of fibroblast growth factors and their receptors in acquired immunodeficiency syndrome-associated Kaposi sarcoma tissue and derived cells. *Cancer*, **72**, 2253–2259.

LISITSYN N, LISITSYN N AND WIGLER M. (1993). Cloning the differences between two complex genomes. *Science*, **259**, 946–951.

LUNARDI-ISKANDER Y, GILL P, LAM VH, ZEMAN RA, MICHAELS F, MANN DL, REITZ MS, KAPLAN M, BERNEMAN ZN, CARTER D, BRYANT JL AND GALLO RC. (1995*a*). Isolation and characterisation of an immortal neoplastic cell line (KS Y-1) from AIDS-associated Kaposi's sarcoma. *J. Natl Cancer Inst.*, **87**, 974–981.

LUNARDI-ISKANDER Y, BRYANT JL, ZEMAN RA, LAM VH, SAMANIEGO F, BESNIER JM, HERMANS P, THIERRY AR, GILL P AND GALLO RC. (1995*b*). Tumorigenesis and metastasis of neoplastic Kaposi's sarcoma cell line in immunodeficient mice blocked by a human pregnancy hormone. *Nature*, **375**, 64–68.

LUPPI M, BARROZI P, MAIORANA A, ARTUSI T, TROVATO R, MARASCA R, CECCHERINI-NELLI L AND TORELLI G. (1995). Herpesvirus-like DNA (KSHV) sequencies in HIV negative reactive lymphadenopathies. *AIDS Res. Hum. Retrovir.*, **11**, S68.

MATHUR WAUGH U, MILDVAN D AND SENIE R. (1985). Follow up at 4 1/2 years on homosexual men with generalised lymphadenopathy. *N. Engl. J. Med.*, **313**, 1542–1543.

MOORE PS AND CHANG Y. (1995). Detection of herpesvirus-like DNA sequences in Kaposi's sarcoma in patients with and without HIV infection. *N. Engl. J. Med.*, **332**, 1181–1185.

NADOR R, CESARMAN E, SAID J, DAWSON B, AOZASA K AND KNOWLES D. (1995). Kaposi's sarcoma herpes virus-like (KSHV) DNA sequences in AIDS and non-AIDS related lymphomas. *AIDS Res. Hum. Retrovir.*, **11**, S68.

NAKAMURA S, SALAHUDDIN SZ, BIBERFELD P, ENSOLI B, MARKHAM PD, WONG SF AND GALLO RC. (1988). Kaposi's sarcoma cells: long-term culture with growth factor from retrovirus-infected CD4+ T cells. *Science*, **242**, 426–430.

PENN I. (1983). Kaposi's sarcoma in immunosuppressed patients. *J. Clin. Lab. Immunol.*, **12**, 1–10.

POLK BF, FOX R, BROOKMEYER R, KANCHANARAKSA S, KASLOW R, VISSCHER B, RINALDO C AND PHAIR J. (1987). Predictors of the acquired immunodeficiency syndrome developing in a cohort of seropositive homosexual men. *N. Engl. J. Med.*, **316**, 61–66.

RABKIN C, BEDI G, MUSABA E, SUNKUTU R, MWANSA N, SIDRANSKY D AND BIGGAR R. (1995*a*). AIDS-related Kaposi's sarcoma is a clonal neoplasm. *Clin. Cancer Res.*, **1**, 257–260.

RABKIN C, BIGGAR R, COLEMAN A, MUSABA E, CHIBWE G AND JANZ S. (1995*b*). Clonality of AIDS-related Kaposi's sarcoma *AIDS Res. Hum. Retrovir.*, **11**, S74.

RADY P, YEN A, ROLLEFSON J, ORENGO I, BRUCE S, HUGHES T AND TYRING S. (1995). Herpesvirus-like DNA sequences in non-Kaposi's sarcoma skin lesions of transplant patients. *Lancet*, **345**, 1339.

RENNE R, ZHONG W, HERNDIER B, MCGRATH M, ABBEY N, KEDES D AND GANEM D. (1996). Lytic growth of Kaposi's sarcoma-associated herpesvirus (human herpesvirus 8) in culture. *Nature Med.*, **2**, 342–346.

SAFAI B, MIKE V, GIRALDO G, BETH E AND GOOD RA. (1980). Association of Kaposi's sarcoma with second primary malignancies: possible etiopathogenic implications. *Cancer*, **45**, 1472–1479.

SALAHUDDIN SZ, NAKAMURA S, BIBERFELD P, KAPLAN MH, MARKHAM PD, LARSSON L AND GALLO RC. (1988). Angiogenic properties of Kaposi's sarcoma-derived cells after long-term culture in vitro. *Science*, **242**, 430–433.

SAMANIEGO F, BRYANT J, ZEMAN R, THIERRY A, JUDDE J, GALLO R AND LUNARDI-ISKANDER Y. (1995). Human Chorionic Gonadotrophin (hCG) prevents development of Kaposi's sarcoma (AIDS KS-Y1) tumours by inducing apoptosis. *AIDS Res. Hum. Retrovir.*, **11**, S76.

SCHALLING M, EKMAN M, KAAYA E, LINDE A AND BIBERFELD P. (1995). A role for a new herpes virus (KSHV) in diferent forms of Kaposi's sarcoma. *Nature Med.*, **1**, 707–708.

SCHULZ TF AND WEISS RA. (1995). Kaposi's sarcoma. A finger on the culprit. *Nature*, **373**, 17–18.

SOULIER J, GROLLET L, OKSENHENDLDER E, CACOUB P, CAZALS-HATEM D, BABINET P, D'AGAY M, CLAUVEL J, RAPHAEL M, DEGOS L AND SIGAUX F. (1995). Kaposi's sarcoma-associated Herpesvirus-like DNA sequences in multicentric Castleman's disease. *Blood*, **86**, 1276–1280.

SU I, HSU Y, CHANG Y AND WANG I. (1995). Herpesvirus-like DNA sequence in Kaposi's sarcoma from AIDS and non-AIDS patients in Taiwan. *Lancet*, **345**, 722–723.

VOGEL J, HINRICHS SH, REYNOLDS RK, LUCIW PA AND JAY G. (1988). The HIV tat gene induces dermal lesions resembling Kaposi's sarcoma in transgenic mice. *Nature*, **335**, 606–611.

VON OVERBECK J, PEREY L, ITEN A, PECHERE M, RUFFIEUX P AND HIRSCHEL B. (1995). Human chorionic gonadotrophin for AIDS-related Kaposi's sarcoma. *Lancet*, **346**, 642–643.

WAHMAN A, MELNICK SL, RHAME FS AND POTTER JD. (1991). The epidemiology of classic, African, and immunosuppressed Kaposi's sarcoma. *Epidemiol. Rev.*, **13**, 178–199.

WALTS A, SHINTAKU I AND SAID J. (1990). Diagnosis of malignant lymphoma in effusions from patients with AIDS by gene rearrangement. *Am. J. Clin. Pathol.*, **94**, 170–175.

WHITBY D, HOWARD M, TENANT-FLOWERS M, BRINK N, COPAS A, BOSHOFF C, HATZIOANNOU T, SUGGETT F, ALDAM D, DENTON A, MILLER R, WELLER I, WEISS R, TEDDER R AND SCHULZ T. (1995). Detection of Kaposi's sarcoma associated herpesvirus in peripheral blood of HIV-infected individuals and progression to Kaposi's sarcoma. *Lancet*, **346**, 799–802.

ZIEGLER JL, KATONGOLE ME, WABINGA H AND DOLLBAUM CM. (1995). Absence of sex-hormone receptors in Kaposi's sarcoma. *Lancet*, **345**, 925.